# Biodegradable Films with Polysaccharides, Proteins, and Bioactive Compounds from *Lobosphaera* sp.: Antioxidant and Antimicrobial Activities

**DOI:** 10.3390/foods14081327

**Published:** 2025-04-11

**Authors:** Valter F. R. Martins, Ana I. Lopes, Manuela Machado, Eduardo M. Costa, Tânia B. Ribeiro, Fátima Poças, Manuela Pintado, Rui M. S. C. Morais, Alcina M. M. B. Morais

**Affiliations:** CBQF—Centro de Biotecnologia e Química Fina—Laboratório Associado, Escola Superior de Biotecnologia, Universidade Católica Portuguesa, Rua Diogo Botelho, 1327, 4169-005 Porto, Portugal; vfmartins@ucp.pt (V.F.R.M.); anlopes@ucp.pt (A.I.L.); mmachado@ucp.pt (M.M.); emcosta@ucp.pt (E.M.C.); tribeiro@ucp.pt (T.B.R.); fpocas@ucp.pt (F.P.); mpintado@ucp.pt (M.P.)

**Keywords:** microalgae, *Lobosphaera* sp., films, polysaccharides, proteins, bioactive compounds, antioxidant activity, antimicrobial activity, cytotoxicity, water vapor permeability

## Abstract

Microalgae are a sustainable source of bioactive compounds and nutrients that do not compete with crops for arable land. *Lobosphaera* sp. was used to produce biodegradable films. Bioactive compounds, polysaccharides, and proteins were extracted from this microalga. The total phenolic content (TPC) and antioxidant activity (ABTS, DPPH, and ORAC) of the bioactive-rich extract were determined, and its composition was analyzed for phenolics using LC-ESI-QqTOF-HRMS and for lipids using GC-FID. The cytotoxicity of this extract on Caco-2 cells was also assessed. Different types of films were produced based on alginate (2%) (film A) and alginate with polysaccharides-rich (PS-rich) extract (0.5%) (film B); PS-rich extract and bioactive-rich extract (0.25%) (film C); protein-rich (P-rich) extract (0.5%) (film D); and P-rich extract and bioactive-rich extract (film E). The antioxidant activity and physical parameters of the films, such as thickness, color, water vapor permeability, solubility, tensile strength (TS), and elongation at break (EAB), were determined. The TPC of the bioactive-rich extract was 1.07 ± 0.05 mg GAE/100 mg DW, and its antioxidant activity was 2.44 ± 0.27, 1.67 ± 0.15, and 11.90 ± 1.22 µmol TE/100 mg DW for ABTS, DPPH, and ORAC, respectively. The extract showed no cytotoxicity to gut cells at concentrations equal to or below 1.0 mg/mL. Film E obtained the best results for the antioxidant activity, 451.06 ± 14.68 and 212.81 ± 39.12 µM TE/mg film for ABTS and DPPH, respectively. In addition, the films enriched with the bioactive-rich extract (films C and E) presented antimicrobial activity against *Listeria monocytogenes*. These films controlled the mold and yeast growth in strawberries during a four-day storage at 25 °C. All films were completely soluble in water and hydroethanolic solutions but only partially solubilized in acetic acid (3%). TS and EAB were not significantly different among the films. It was possible to produce biodegradable films using microalga *Lobosphaera* sp. with good bioactivity and physical characteristics.

## 1. Introduction

Microalgae are explored for their production of secondary metabolites, notably antimicrobial and antioxidant compounds, which hold significant potential for application in food products [1]. While microalgae are rich in lipids, proteins, and carbohydrates, their compositional profiles can vary significantly across different species and strains, posing challenges to their consistent utilization. *Lobosphaera* sp. is an oleaginous green microalga predominantly found in freshwater environments [2], although it can also be found in marine environments [1]. It is recognized for its production of bioactive compounds, including carotenoids and polyunsaturated fatty acids (PUFAs). *Lobosphaera bisecta* has been identified as a significant producer of arachidonic acid, comprising up to 47% of its total fatty acids and accounting for 54% of its biomass. *Lobosphaera* (formerly *Parietochloris*) *incisa* is a notable producer of dihomo-*γ*-linolenic acid (DGLA), which can comprise up to 12.3% of its dry weight [3]. The metabolism of arachidonic acid (ARA) under inflammatory conditions influences macrophage immune reactivity and modulates key inflammatory pathways in various models. This modulation plays a potential role in preventing Alzheimer’s disease, making it of significant biotechnological interest [4,5]. In dietary studies, feeding *Lobosphaera incisa* to zebrafish (*Danio rerio*) was shown to increase fatty acid content in the gut, particularly ARA, thereby enhancing immunomodulatory functions, such as improved resistance to streptococcal infection [6]. In food applications, microalgae are gaining attention as a sustainable alternative to fish and plant-based oils due to their ability to accumulate substantial amounts of eicosapentaenoic acid (EPA) and docosahexaenoic acid (DHA) [7].

The incorporation of microalgal biomass into film matrices has been explored to enhance film properties. For instance, studies have demonstrated that integrating microalgae such as *Heterochlorella luteoviridis* and *Dunaliella tertiolecta* into cassava starch films can modify mechanical and barrier characteristics, leading to increased solubility, biodegradability, and opacity, while reducing flexibility and hydrophilicity. Notably, these microalgae-enriched films exhibited enhanced antioxidant activity [8]. Morales-Jimenez et al. [9] developed films using extracellular biopolymers derived from the spent culture media of *Nostoc* sp. and *Porphyridium purpureum*. These films exhibited strong antifungal activity against *Fusarium verticillioides* and *Fusarium* sp. However, they presented microscopic fissures. Agustini et al. [10] formulated an edible film by incorporating *Chlorella vulgaris* ethanolic and water extracts into a cornstarch-based matrix. The film effectively extended its shelf life when applied to *dodol*, an Indonesian glutinous rice cake prone to rancidity.

This study focuses on extracting bioactive compounds, polysaccharides, and proteins from *Lobosphaera* sp. microalga to produce films for packaging, analyzing the extracts, and determining the film’s physical and antioxidant properties. These films could increase the shelf life of foods while being biodegradable, and the produced films were tested on packed strawberries stored at 25 °C.

## 2. Materials and Methods

### 2.1. Microalga Biomass

*Lobosphaera* sp. biomass *Arthospira* sp. biomass was kindly donated frozen by A4F—Algae for Future, Portugal. It was stored at −20 °C until it was necessary for the extraction assays of bioactive compounds, polysaccharides, and protein, at which point it was freeze-dried.

### 2.2. Extraction of Bioactive Compounds from Lobosphaera sp.

A classical solvent extraction method was employed using a hydroalcoholic solution (water/ethanol, 1:9 *v*/*v*). One gram of *Lobosphaera* sp. powder was immersed in 30 mL of the solution and incubated at 50 °C with continuous agitation at 120 rpm (Orbital Shaker, MaxQ 6000, Thermo Scientific, Waltham, MA, USA) for 120 min, with the process repeated twice. The mixture was then subjected to ultrasonic homogenization using a probe sonicator (Sonics, Vibra-Cell, Newtown, CT, USA) with 20 kHz pulses applied for 30 s at a time over a duration of 10 min. Following homogenization, the solution was filtered, and the ethanol was removed through rotary evaporation (Buchi R-210, Buchi Labortechnik AG, Flawil, Switzerland). Finally, the extract was lyophilized to obtain a dry powder [11].

The extraction yield was measured, and the resulting bioactive-rich extract was utilized for film production (Section 2.9). The remaining residue from this process was further used to extract polysaccharides (Section 2.7) and proteins (Section 2.8).

### 2.3. Identification of Phenolic Compounds in Lobosphaera sp. Extracts

The *Lobosphaera* sp. extract was analyzed for phenolic compounds using liquid chromatography coupled with electrospray ionization quadrupole time-of-flight high-resolution mass spectrometry (LC-ESI-QqTOF-HRMS, Impact II, Bruker Daltonics, Bremen, Germany), based on Vilas-Boas et al. [12]’s methodology with modifications. The extract (50 mg/mL) was dissolved in ultrapure water, followed by the addition of 1 mL of ice-cold methanol (−80 °C) to precipitate proteins.

The chromatographic separation was carried out on a UHPLC UltiMate 3000 Dionex system (Thermo Fisher Scientific Inc., Waltham, MA, USA), equipped with an ultra-high-resolution QqTOF mass spectrometer (Impact II, Bruker Daltonics, Bremen, Germany) offering a full-sensitivity resolution of 50,000 FSR. The separation utilized an Acclaim RSLC 120 C18 column (100 mm × 2.1 mm, 2.2 µm) (Thermo Fisher Scientific Inc., Sunnyvale, CA, USA). Compound identification was achieved through comparison of retention times and mass spectra with those of standard solutions when available. For unidentified peaks, tentative identifications were made based on the literature, with elemental compositions confirmed via precise mass measurements (within ±5 mDa) and mSigma values < 20. A single independent analysis was carried out for each of the triplicate extracts.

### 2.4. Identification of Fatty Acids Profile in Lobosphaera sp. Extracts

The gas chromatography with flame ionization detector (GC-FID) analysis was performed, following Machado et al. [13,14] method with some modifications. The fatty acids profile of the microalgae extracts (Section 2.2) was determined after transesterification. Briefly, 250 mg of the sample was added to 100 μL of tritridecanoin (1.5 mg/mL). Then, 900 μL of hexane and 2.25 mL of methanol were added, followed by 240 μL of sodium methoxide (5.4 M). The samples were vortexed and incubated at 80 °C for 10 min. They were then cooled on ice before adding 1.25 mL of DMF and 1.25 mL of 3 M sulfuric acid in methanol. After vortexing, the samples were incubated at 60 °C for 30 min. Once cooled, they were vortexed again and centrifuged at 1250× *g* for 5 min at 18 °C. The upper layer was then collected for further analysis.

Gas chromatography (GC) analyses were conducted using an Agilent 8860 gas chromatograph (Agilent, Santa Clara, CA, USA) equipped with a flame ionization detector (FID) and a BPX70 capillary column (60 m × 0.25 mm, 0.25 µm; SGE Europe Ltd., Paris, France). The operating conditions were as follows: the injector (split ratio of 25:1, injection volume 1 μL) was set at 250 °C, while the FID temperature was maintained at 275 °C. The carrier gas was hydrogen at a flow rate of 1 mL/min (20.5 psi). The oven temperature was programmed to start at 60 °C (held for 5 min), increase at a rate of 15 °C/min to 165 °C (held for 1 min), and then rise at 2 °C/min to reach 225 °C (2 min). Fatty acid identification was carried out using Supelco 37 standards. Each GC analysis was performed in triplicate. In addition, the nutritional quality of the extracts was determined by the following indexes:

Index of atherogenicity (AI)

(1) $$AI=\frac{\left[C12:0+4\times \left(C14:0\right)+C16:0\right]}{\left(\Sigma MUFA+\Sigma PUFA\;n6+\Sigma PUFA\;n3\right)}$$

Index of thrombogenicity (TI)

(2) $$TI=\frac{\left(C14:0+C16:0+C18:0\right)}{\left[0.5\times \Sigma MUFA+0.5\times \Sigma PUFA\;n6+3\times \Sigma PUFA\;n3+\left(\frac{\Sigma PUFAn3}{\Sigma PUFAn6}\right)\right]}$$

Hypocholesterolemic-to-Hypercholesterolemic ratio (HH)

(3) $$HH=\frac{\left(cis-C18:1+\Sigma PUFA\right)}{\left(C12:0+C14:0+C16:0\right)}$$

Health-Promoting Index (HPI)

(4) $$HPI=\frac{\Sigma UFA}{\left[C12:0+\left(4\times C14\right)+C16\right]}$$

MUFA—monounsaturated fatty acids; PUFA—polyunsaturated fatty acids, UFA—unsaturated fatty acids, PUFA n3—ω-3 polyunsaturated fatty acids, PUFA n6—ω-6 polyunsaturated fatty acids. C12:0—lauric acid; C14:0—myristic acid; C16:0—palmitic acid; and C18:0—stearic acid.

### 2.5. Cellular Cytotoxicity Analysis of Lobosphaera sp. Extracts

The cytotoxicity evaluation of the freeze-dried extracts, prepared under the extraction conditions detailed in Section 2.2, was conducted in accordance with the ISO 10993-5:2009 standard [15]. In brief, Caco-2 cells (ATCC HTB-37) were cultured until reaching 80–90% confluence, then detached using TrypLE™ Express (Thermo Fisher Scientific, Waltham, MA, USA) and seeded into 96-well microplates at a density of 1 × 10^4^ cells per well. After 24 h, the culture medium was replaced with a medium containing the freeze-dried extracts at different concentrations. A 30% (*v*/*v*) dimethyl sulfoxide (DMSO) solution (Sigma, St. Louis, MO, USA) was used as a cytotoxicity control, while untreated medium served as the cell viability control.

Following 24 h of incubation, PrestoBlue™ reagent (Thermo Fisher Scientific, Waltham, MA, USA) was added to each well, and the microplates were incubated for an additional hour. Fluorescence was then measured in a microplate reader (Synergy H1, BioTek Instruments, Winooski, VT, USA) at an excitation wavelength of 560 nm and an emission wavelength of 590 nm. The cytotoxicity results were expressed as the percentage of metabolic inhibition relative to the cytotoxic control. The experiments were conducted in quadruplicate.

### 2.6. Characterization of Bioactivity of Lobosphaera sp. Extracts

#### 2.6.1. Total Phenolic Content (TPC)

The total phenolic content (TPC) was determined using the Folin–Ciocalteu method, following the procedure outlined by Martins et al. [11]. Absorbance was measured at 765 nm using a Synergy H1 microplate reader (BioTek, Winooski, VT, USA) in a 96-well microplate (Sarstedt, Nümbrecht, Germany). The standard used for the calibration curve was gallic acid, and the results were expressed as mg GAE/mg DW. Each sample was analyzed in triplicate, with three independent assessments performed.

#### 2.6.2. Antioxidant Activity

The antioxidant activity of the extract solutions (20 mg/mL) was accessed using three different assays: ABTS, DPPH, and ORAC.

The ABTS (2,2′-azinobis(3-ethylbenzothiazoline-6-sulfonic acid) assay, DPPH (2,2-diphenyl-1-picrylhydrazyl) assay, and ORAC (oxygen radical absorbance capacity) assay were performed according to the methodology described by Martins et al. [11]. Trolox was used as the standard for the calibration curve, and the results were expressed as µmol TE/100 mg DW. Each sample was analyzed in triplicate, with three independent assessments conducted.

### 2.7. Extraction of Polysaccharides from Dried Lobosphaera sp.

The solid-phase residue (Section 2.2) was dispersed in distilled water at a concentration of 50 g/L and heated to approximately 90 °C with continuous magnetic stirring (500 rpm) using an IKA RCT basic (Staufen, Germany) for 60 min. The resulting solution was filtered through filter paper, and the process was repeated.

Polysaccharides (PS) were then precipitated by adding ethanol until a 50:50 water-to-ethanol ratio was achieved, followed by centrifugation at 5000 rpm for 20 min. The precipitate was re-dissolved in distilled water and subsequently freeze-dried [16]. The extraction yield was determined, and the PS-rich extract was used for the production of films (Section 2.9).

### 2.8. Extraction of Protein from Lobosphaera sp.

Martins et al. [17]’s methodology was used to obtain a protein-rich extract from freeze-dried *Lobosphaera* sp. Initially, acid hydrolysis was performed using 2% acetic acid at 50 °C, with a solid-to-liquid ratio of 1:3 (*w*/*v*), under agitation at 125 rpm for 1 h. An enzymatic hydrolysis with cellulase (5%) at 50 °C and pH of 7.5, with a ratio of 1:10 (*w*/*v*) at 125 rpm for 2 h, was performed. Subsequently, protease (3.9%) was used for a secondary hydrolysis step at 40 °C and pH 7.5 under the same agitation and duration. Enzymatic activity was terminated by heating the mixture at 90 °C for 10 min. The resulting mixture was then centrifuged at 5000× *g* for 20 min. The supernatant, containing the bioactive extract enriched with protein hydrolysates, was subsequently freeze-dried.

### 2.9. Film Production Using the “Casting” Method

Films were prepared following Martins et al. [16]’s methodology, with some modifications. Five different types of films were developed (Table 1): a control film containing only alginate (Film A), two films incorporating a polysaccharide-rich (PS-rich) extract—one with alginate and PS-rich extract (Film B) and another with alginate, PS-rich extract, and bioactive-rich extract (Film C). Additionally, two films were formulated with a protein-rich (P-rich) extract—one with alginate and P-rich extract (Film D) and another with alginate, P-rich extract, and bioactive-rich extract (Film E).

To prepare the film-forming solution, sodium alginate (2% *w*/*v*) (Sigma, Aldrich Chemie GmbH, Steinheim, Germany) was dissolved in hot distilled water. Either 0.5% *w*/*v* of the PS-rich extract (Section 2.7) or the P-rich extract (Section 2.8) was then added and stirred until fully dissolved, followed by continuous stirring for one hour. Subsequently, diacetyllauroyl glycerol (0.6% *v*/*v*) (Tokyo Chemical Industry, Toshima, Kita-ku, Tokyo, Japan) was incorporated into the alginate and PS or P solution and stirred for another hour at room temperature (25 °C). For films enriched with the bioactive extract from *Lobosphaera* sp., the bioactive-rich extract (0.25% *w*/*v*) was added under stirring until completely dissolved.

To form the films, 20 mL of the prepared solution was poured into a 10 cm diameter plastic Petri dish and dried at 40 °C for 18 h in an oven (Memmert GmbH, Schwabach, Germany). Alginate was selected as the primary film-forming agent, as polysaccharides extracted from *Lobosphaera* sp. alone do not form a cohesive film [9].

### 2.10. Film Characterization

#### 2.10.1. Determination of Physical Properties of the Film

- Thickness

A digital thickness gauge (Adamel Lhomargy, Ivry-sur-Seine, France) was used to measure the thickness of the films. Three replicates were prepared for each sample, and five random thickness measurements were taken per replicate [17].

- Color

The color of each film was analyzed using a Chroma Meter CR 400 (Konica Minolta Sensing, Osaka, Japan), which was calibrated with a white standard color plate. Color measurements were recorded in the L*, a*, and b* system [18]. Hue and Chroma values were calculated using the following equations:

Hue = arctan(b*/a*) (5)

(6) $$Chroma=\sqrt{{a*}^{2}+{b*}^{2}}$$

- Water vapor permeability

The water vapor transmission rate (WVTR) and water vapor permeability (WVP) of the films were determined gravimetrically at 23 ± 3 °C and 50% relative humidity (RH), as mentioned by Martins et al. [17]. Briefly, the procedure involved placing dried calcium carbonate in a capsule, sealing the capsule with the film, and weighing it twice a day. The WVTR (g·m^−2^·day^−1^) and WVP (g·mm·m^−2^·day^−1^·kPa^−1^) were calculated through linear regression and by applying the following formulae:

WVTR = weight of water that passed through the film/(area × time)(7)

WVP = WVTR × thickness of the film/(water vapor saturation pressure × ∆RH)(8)

- Solubility

The film solubility was determined using the same food simulants foreseen to test migration into different foods, according to the regulation EU N°10/2011 [19]. The solubility of the films in water and four solutions, water and ethanol (10 and 20% *v*/*v*) mimicking hydrophilic foods, water and ethanol 50% *v*/*v* solutions mimicking lipophilic foods (e.g., dairy foods), and acetic acid (3%) mimicking foods with pH lower than 4.5 was tested. The solutions were prepared, and the films were maintained in water or each solution for 24 h at room temperature (ca. 15 °C). The solubility was determined using the following equation:

Solubility (%) = 100 − film weight after 24 h immersion/film initial weight × 100(9)

- Tensile strength (TS) and elongation at break (EAB)

Tensile strength (TS) and elongation at break (EAB) were determined using a Texture Analyzer (Stable Micro Systems, model TA.XT.Plus, Godalming, UK) equipped with mini tensile grips and a 5000 g load cell.

The films were conditioned at 23 °C and 50% relative humidity for 48 h following the ASTM D-882 standard [20]. The films were then cut into rectangular strips of 6 cm and 2 cm (length × width) and analyzed. Each film was carefully mounted between the upper and lower tensile grips. The test was conducted at a crosshead speed of 0.5 mm/s until film rupture. The stress (MPa) and strain (%) were recorded during the test. The graphical representation of these parameters allowed for the determination of TS (*y*-axis, stress) and EAB (*x*-axis, strain). These values corresponded to the highest point of the curve, indicating the film breaking point. The results were calculated as the average of five film samples.

#### 2.10.2. Determination of the Film Antioxidant Activity (ABTS, DPPH)

The antioxidant activity of each film was assessed using the ABTS (2,2′-azinobis(3-ethylbenzothiazoline-6-sulfonic acid)) and DPPH (2,2-diphenyl-1-picrylhydrazyl) scavenging assays, following the method described by Lopes et al. [21]. In brief, the ABTS solution was adjusted with water to achieve an initial absorbance of 0.700 ± 0.020 at 734 nm (Synergy H1, Biotek, Winooski, VT, USA). A 90 µM DPPH working solution was prepared with methanol to reach an absorbance of 0.600 ± 0.100 at 515 nm.

For each film, a piece (1 mg) was placed in a test tube, and 2–8 mL of the ABTS or DPPH solution was added. The tubes were kept protected from light and maintained at room temperature (25 °C). The ABTS solution reacted for 6 min, while the DPPH solution reacted for 30 min. After the reaction times, absorbance was measured at 734 nm for ABTS and 515 nm for DPPH. Results were expressed as micromoles of Trolox equivalents per milligram of film (µM TE/mg film). All analyses were performed in quadruplicate.

#### 2.10.3. Determination of the Film Antimicrobial Activity

The antibacterial activity of the films was evaluated using the viable cell method, as described by Lopes et al. [21]. Prior to testing, an overnight liquid culture of the selected bacterial strain was grown in Mueller–Hinton broth (Biokar Diagnostics, Beauvais, France), and the optical density was adjusted to 0.2 at 610 nm, corresponding to approximately 10^8^ CFU/mL. The culture was then diluted in the same broth to achieve an inoculum concentration of 10^5^–10^6^ CFU/mL.

The films were cut into 1 cm disks (approximately 10 mg) and sterilized by exposure to UV light (254 nm) for 10 min on each side. The sterilized film disks were then placed into sterile tubes, and 200 µL of the inoculum was added to each tube. The films were incubated at 37 °C for various time intervals (0, 4, 8, 13, 24 h). Peptone water (1.8 mL) was then added to each tube, and the solution was homogenized until complete dissolution of the film disk. Four ten-fold serial dilutions of each solution were made in sterile peptone water. Aliquots (20 µL) from each dilution were plated onto Mueller–Hinton agar plates, which were then incubated at 37 °C for 24 h. Colony counts were recorded after the incubation period.

The results were expressed as log(CFU/mg of film), with the assay’s detection limit set at 1.6 log(CFU/mg of film). Two replicates of each film were tested at each time point, and each dilution was plated in duplicate. Therefore, the reported results represent the average of four colony counts per film per time point.

The biological material (bacteria) used in the tests was sourced from the CBQF collection, which included isolates from rabbits and human clinical samples. The species tested were *Salmonella enterica serovar* Enteritidis ATCC 13076 and *Listeria monocytogenes* ESB 3562.

### 2.11. Film Application on Strawberry

Organic strawberries (*Fragaria x ananassa*) with similar dimensions were purchased from the local market. To prepare strawberries before film application, they were rinsed with 50 ppm chlorinated water for 3 min, followed by immersion in distilled water for 1 min and subsequently drained. Each treated strawberry (26.53 ± 4.40 g) was then placed in a sterile 100 mL flask, which was sealed with the film (25.5 cm^2^). Three replicates were performed for each film.

The flasks were stored at room temperature (approximately 25 °C) for four days. The weight of each strawberry was registered each day, and the moisture loss was calculated. At the end of the storage microbiological analyses were performed.

#### 2.11.1. Moisture Loss

The moisture loss was determined using the formula:

Moisture loss = ((initial weight (day 0) − current weight (day D))/initial weight (day 0)) × 100(10)

#### 2.11.2. Microbiological Analyses

At the end of the storage, the strawberries were analyzed for mesophilic aerobic bacteria, molds, and yeasts. These microbiological analyses followed Moreira et al.’s [22] methodology. Approximately 10 g of strawberry from each sample was macerated in 90 mL of 1 g/L peptonized water and homogenized using a Stomacher homogenizer (Seward West Sussex, UK). Serial 1:10 dilutions of each homogenized sample were prepared and plated in duplicate. Enumeration and differentiation of microorganisms were conducted using the appropriate media and conditions. Mesophilic aerobic bacteria were cultured on Plate Count Agar (PCA) and incubated at 30–32 °C for 48–72 h. Molds and yeasts were quantified on Potato Dextrose Agar (PDA) and incubated at 25 °C for five days. Microbial counts were performed in duplicate across three independent experimental replicates.

### 2.12. Statistical Analysis

The results were expressed as the mean ± standard deviation of three independent replicates (n = 3). The Shapiro–Wilk test was used to assess normality, and the Levene test was performed to evaluate the homogeneity of variance on the residuals of the fitted model. All data followed a normal distribution and were statistically compared (control vs. different formulations) using one-way ANOVA. Tukey’s test (*p* ≤ 0.05) was applied when the variance was homogeneous, and Dunnett’s C test (*p* ≤ 0.05) was used when the variance was heterogeneous. Statistical analysis was performed using SPSS Base 23.0 for Windows (SPSS Inc., Chicago, IL, USA).

## 3. Results

### 3.1. Yield of Extraction of Bioactive Compounds, Polysaccharides, and Proteins from Freeze-Dried Lobosphaera sp.

The hydroethanolic ultrasound-assisted extraction of bioactive compounds from *Lobosphaera* sp. yielded 15.48 ± 1.35% dry weight (DW). The extraction yields for polysaccharides (PS) and protein (P) were 1.85 ± 0.02% DW and 30.34 ± 4.79% DW, respectively.

Monteiro et al. [23] also performed ultrasound-assisted extraction of bioactive compounds using hydroalcoholic solutions containing 50% and 80% ethanol, obtaining yields of 17.33 ± 1.00% and 24.44 ± 1.88%, respectively, for *Nannochloropsis* sp. The same method applied to *Chlorella* sp. resulted in higher yields of 26.72 ± 1.74% and 28.05 ± 2.09%, respectively. Similarly, Martins et al. [17] reported even greater extraction efficiencies for *Arthrospira* sp., with polysaccharide extraction yielding 7.841 ± 0.255% DW and protein extraction yielding 46.07 ± 4.22% DW.

### 3.2. Phenolic Compounds in Lobosphaera sp. Bioactive-Rich Extract

No phenolic compounds were detected in the bioactive-rich extract of the present study using LC-ESI-QqTOF-HRMS. This absence may be attributed to a very low concentration of phenolics in *Lobosphaera* sp., their potential oxidation prior to analysis, or the presence of high-molecular-weight phenolic compounds. Given that the detection limit of LC-ESI-QqTOF-HRMS is below 2000 Da, larger phenolic compounds may have remained undetected.

No studies on the phenolic profile of *Lobosphaera* sp. were found in the literature. However, within the division (phylum) *Chlorophyta*, which includes *Lobosphaera* sp., Goiris et al. [24] identified several phenolic compounds—such as phloroglucinol, p-coumaric acid, ferulic acid, and apigenin—in *Tetraselmis* and *Chlorella* using UHPLC-MS/MS. Goiris et al. [25] further refined their analysis by treating microalgal extracts from different phyla with ion exchange chromatography to remove pigments and other compounds. Using UHPLC-MS/MS, they detected only sub-microgram levels of phenolic acids and flavonoids. Sozmen et al. [26] analyzed *Chlorella miniata* and identified ~70 μg/g DW of trans-cinnamic acid and <2.5 μg/g DW of catechin. Salicylic and caffeic acids were present at significantly higher concentrations, with salicylic acid reaching ~650 μg/g DW and caffeic acid exceeding 1 mg/g DW. Safafar et al. [26] noted that some studies suggest the phenolic content in microalgae is comparable to or lower than the minimum levels reported for terrestrial plants. Using HPLC, they identified caffeic acid, ferulic acid, *p*-coumaric acid, and cinnamic acid in *Chlorella sorokiniana*. Beyond the *Chlorophyta* division, various phenolic compounds have been detected in other microalgae species, including *Arthrospira* sp., *Nostoc* sp., *Nannochloropsis* sp., and *Anabaena doliolum*. Among the identified compounds were gallic acid, salicylic acid, kaempferol, quercetin, luteolin, and naringenin [24,27].

Conversely, some studies have also failed to detect phenolic compounds in various microalgal biomasses and extracts. Parkes et al. [28] did not identify any reference phenolics in *Stauroneis* sp. (a diatom) or *Tetraselmis chuii* (a green microalga like *Lobosphaera* sp.) using HPLC-U. Similarly, Andriopoulos et al. [29] detected no phenolics in extracts of *Chlorella minutissima*, *Dunaliella salina*, *Tisochrysis lutea*, *Isochrysis galbana*, or *Nannochloropsis oculata* using LC-MS. Bernard and Guéguen [30] also reported the absence of nine standard phenolic compounds in *Euglena gracilis* when analyzed using HPLC-Q-TOF-MS.

### 3.3. Fatty Acids in Lobosphaera sp. Bioactive-Rich Extract

The bioactive-rich extract from *Lobosphaera* sp. contained high concentrations of palmitic acid, followed by oleic acid, γ-linolenic acid (ω-6), and α-linolenic acid (ω-6) (Table 2). Additionally, eicosapentaenoic acid (EPA) and docosahexaenoic acid (DHA)—both commonly found in this microalga—were also present in the extract (Table 2).

*Lobosphaera incisa* accumulates a high percentage (60% of total fatty acids) of the essential ω-6 LC-PUFAs, including arachidonic acid, in its triacylglycerols [2,31]. The hydroalcoholic extract from *Lobosphaera* sp. also contained a significant amount of ω-6 LC-PUFAs; however, arachidonic acid was present at lower concentrations in the extract of the present study (Table 2).

Dietary fats, primarily composed of triglycerides and fatty acids, play either positive or negative roles in the prevention and treatment of diseases. Several indices are used to assess the nutritional quality of fats, including the Atherogenicity Index (AI), Thrombogenicity Index (TI), Hypocholesterolemic-to-Hypercholesterolemic ratio (HH), and Health-Promoting Index (HPI). These indices provide valuable information about the nutritional value of extracts. The AI reflects the ratio between the sum of saturated fatty acids (SFAs) and unsaturated fatty acids (UFAs). UFAs are considered antiatherogenic. Therefore, consuming foods or products with a low AI can help decrease total cholesterol and LDL-C levels in human blood plasma. In this study, the AI of *Lobosphaera* sp. extract was 0.890 ± 0.123, which is within the range observed for seaweeds, from 0.03 ± 0.003 for *Gracilaria changii* to 3.06 ± 0.611 for *Lomentaria clavellosa* [31]. The TI characterizes the thrombogenic potential of fatty acids (FAs). It also reflects the contribution of different FAs, highlighting the relationship between pro-thrombogenic FAs (C12:0, C14:0, and C16:0) and antithrombogenic FAs (MUFA and the n3 and n6 types). In a study on *Sargassum* seaweeds, the TI of *Sargassum pallidum* was 1.60 ± 0.56, which was similar to the values of the present study, 1.649 ± 0.206, and TI was used to evaluate the cardiovascular health effects of four *Sargassum* species [32]. The TI for other seaweeds ranged from 0.46 to 1.60. Generally, the TI for seaweeds ranges from 0.04 to 2.94, with the exception of *Gracilaria salicornia*, which had a TI of 5.75 [32]. An extract with a low AI and TI, based on its fatty acid composition, is considered to have better nutritional quality, potentially reducing the risk of coronary heart disease. In this study, the HH ratio was 3.75 ± 0.003, which characterizes the relationship between hypocholesterolemic fatty acids (cis-C18:1 and PUFAs) and hypercholesterolemic fatty acids. For macroalgae, HH values range from 1.26 to 4.22 [32]. The HPI is the inverse of the AI, with higher HPI values indicating foods that are more beneficial to human health. In this study, the HPI for *Lobosphaera* sp. extract was 1.151 ± 0.156, while for dairy products, the HPI ranges from 0.16 for milk to 0.68 for Syrian cheese [32].

### 3.4. Cytotoxicity of Lobosphaera sp. Bioactive-Rich Extract

**Figure 1:**
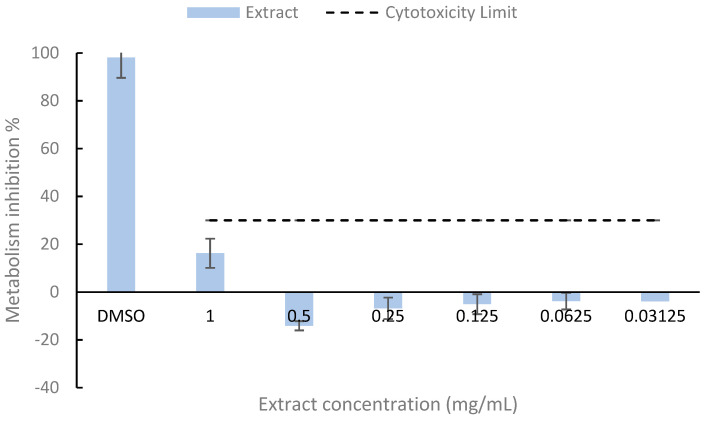
Cytotoxic Effect of a hydroethanolic extract from *Lobosphaera* sp. in cells Caco-2. The dotted line presents the 30% cytotoxicity limit as defined by ISO 10993-5 [15].

The cytotoxicity assay results (Figure 1) indicate that all extract concentrations were below the cytotoxicity threshold, following the ISO 10993-5:2009 standard [15]. Therefore, no cytotoxic effects were observed at any of the tested concentrations.

Although the literature does not report the presence of phycotoxins in *Lobosphaera* sp., Caco-2 cells can serve as a reliable model for cytotoxicity testing [33]. Other studies, such as those by Guo et al. [34], tested water extracts from *Chlorella pyrenoidosa*, *Spirulina platensis*, and *Synechococcus* sp. on Caco-2 cells and found no significant cytotoxicity at a concentration of 8 µg/mL. This concentration is considerably lower than those tested in the present study, further supporting the good biocompatibility of the bioactive-rich extract toward Caco-2 cells.

### 3.5. Total Phenolic Content and Antioxidant Activity of Lobosphaera Bioactive-Rich Extract

The hydroethanolic extracts of *Lobosphaera* sp. showed good antioxidant activity.

Corrêa et al. [35] also investigated the total phenolic content (TPC) and antioxidant activity of ethanolic extracts from *Lobosphaera* sp., examining the effects of high-pressure processing (HPP) and comparing frozen biomass with freeze-dried biomass. Their results show that the highest TPC, 11.50 ± 0.21 mg GAE g^−1^ DW, was obtained when HPP was applied to the frozen biomass. This value is comparable to the 10.7 ± 0.5 mg GAE g^−1^ DW determined in the present study (Table 3).

Torres et al. [36] reported that the phenolic content in red (*Rhodophyta*) and green (*Chlorophyta*) algae is generally lower than in brown algae (*Phaeophyceae*, *Ochrophyta*). They also noted that TPC values can vary depending on the production conditions of the microalgae or the geographic regions where they are collected. However, they observed that the average TPC values in algal biomass were lower than in algal extracts. In the present study, *Lobosphaera* sp., a green microalga, exhibited a TPC slightly lower than 13 mg GAE g^−1^ DW, which is consistent with the reported values for green microalgae [36].

The total phenolic content (TPC) assay using the Folin–Ciocalteu method may be influenced by interfering compounds (non-phenolic substances). It is expected that compounds such as vitamins and their derivatives, inorganic acids, amino acids, or chlorophylls could affect the accurate estimation of TPC in microalgae extracts. For instance, chlorophyll content in microalgae can reach as high as 5%, and its interference is a significant challenge in TPC determination. Additionally, guanine, a major amino acid, is particularly reactive with the Folin–Ciocalteu reagent, with a response of approximately 0.34 mg GAE/mg guanine. The response of aromatic amino acids ranges from 0.28 to 0.41 mg GAE/g [37].

### 3.6. Produced Films

The film produced using the casting method demonstrated good film-forming ability, even when extracts rich in polysaccharides, proteins, and bioactive compounds were incorporated into the alginate-based film.

**Figure 2:**
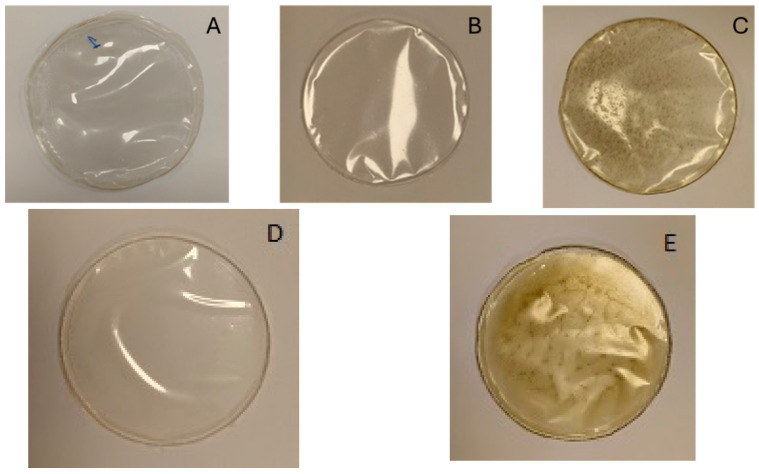
Films with alginate 2% (**A**); alginate 2% + PS-rich extract 0.5% (**B**); alginate 2% + PS-rich extract 0.5% + bioactive-rich extract 0.25% (**C**); alginate 2% + P-rich extract 0.5% (**D**); and alginate 2% + P-rich extract 0.5% + bioactive-rich extract 0.25% (**E**).

Regarding the appearance of the films, the primary difference observed among them was the color. Film A (Figure 2), which consisted solely of alginate (control), closely resembled films B and D in color. However, films C and E were darker, exhibiting a slight brown tint, likely due to the addition of the bioactive extract in the formulation (color results are provided in Section 3.7.1).

### 3.7. Film Properties

#### 3.7.1. Physical Properties

- Thickness and water vapor permeability (WVP)

A significant difference in thickness was observed between film A, which measured 0.046 ± 0.003 mm, and all other films (Table 4). However, the results indicate a trend in which the thickness of the film increased with the addition of the bioactive-rich extract. Additionally, film B exhibited a significantly different thickness compared with film D.

Kontogianni et al. [38] also observed that the thickness of a film containing whey protein and *Spirulina* biomass increased as the concentration of *Spirulina* biomass in the film increased. However, Silva et al. [39] found that an increase in the concentration of *K. alvarezii* powder led to a decrease in film thickness.

The film E exhibited the lowest water vapor permeability (WVP) at 12.27 ± 0.98 g·mm·m^−2^·day^−1^·kPa^−1^, although this value was not significantly different from the films D or C (Table 4). The WVP of the alginate film (23.45 ± 1.47 g·mm·m^−2^·day^−1^·kPa^−1^) was not significantly different from the other films, except for film E, containing protein and bioactive compounds. Martins et al. [17] also observed relatively low WVP values for films containing P-rich extract with or without bioactive-rich extract from *Arthrospira* sp.: 14.39 ± 3.64 g·mm·m^−2^·day^−1^·kPa^−1^ for alginate + P-rich extract film, and 12.28 ± 3.01 g·mm·m^−2^·day^−1^·kPa^−1^ for alginate + P-rich extract + bioactive extract film. The latter value was similar to that found in the present study for the *Lobosphaera* sp. film.

The findings of Mondal et al. [40] align with the results of this study, showing that the WVP value decreased with the addition of algae ethanolic extract (CAEE) to the chitosan film formulation. Furthermore, increasing the CAEE concentration from 4% to 28% resulted in a reduction in WVP values. Carissimi et al. [8] produced films using starch and hydroalcoholic extracts of *Heterochlorella luteoviridis* and *Dunaliella tertiolecta* microalgae biomasses. They observed that films containing microalgae extracts exhibited lower WVP values compared with those containing microalgae biomass. This difference may be attributed to the higher concentration of lipophilic compounds in the microalgae extract films, which could reduce water–starch interactions due to their hydrophobic nature. A similar effect may have occurred with the microalgae extracts and alginate, resulting in a decrease in WVP values. Zinina et al. [41] found that the incorporation of protein hydrolysate (1.5%) significantly increased the thickness of alginate films, suggesting an increase in the dissolved solid content within the matrix. It also resulted in a decrease in the WVTR of the alginate film. Protein hydrolysates, which are combinations of amino acids and peptides, consist of oligopeptides and free amino acids. These bioactive components may interact with the alginate matrix, enhancing the film’s integrity and reducing its permeability.

- Color Parameters

In general, the addition of different extracts to the film formulation resulted in changes to the color parameters. The films C and E, containing bioactive compounds, exhibited the highest b* and Chroma values (Table 5). Wang et al. [42] reported an increase in a* and b* values, and a reduction in L* value in a chitosan-based film after the incorporation of tea polyphenols, compared with the control.

The literature suggests that active films containing naturally colored pigments may contribute to the alteration of color coordinates. For instance, films made by Mondal et al. [40] with chitosan showed a decrease in L* and a* values, alongside an increase in b* values as the concentration of *Dunaliella tertiolecta* ethanolic extract increased. The rise in negative a* values might be attributed to the increasing concentration of chlorophyll or carotenoids in the algal ethanolic extract. Similarly, in the present study, an increase in the b* value was observed when *Lobosphaera* sp. PS- or P-rich extracts were incorporated into the film formulation (Table 5).

- Solubility characteristics

The three types of edible films produced were fully soluble in water and hydroethanolic solutions (10%, 20%, and 50%). However, they were not completely soluble in 3% acetic acid. Significant differences were observed between film A (the control) and film D, containing protein (Table 6). These findings were consistent with those reported for *Arthrospira* sp. films [17], and the solubility of P-rich extract films from *Lobosphaera* sp. in 3% acetic acid was higher compared with P-rich extract films from *Arthrospira* sp. In the acetic acid solution, the P-rich extract films from *Lobosphaera* sp. showed higher solubility than the control alginate films and the PS-rich extract films (Table 6).

It is important to note that films intended for food packaging should not exhibit excessively high solubility, particularly for foods with high moisture content. In contrast, edible coatings require high solubility [43]. Silva et al. [39] observed that solubility increased with higher concentrations of microalgae biomass in the formulation. Additionally, Zinina et al. [41] found that incorporating protein hydrolysate (1.5%) significantly reduced the solubility of the alginate film.

- Mechanical properties

The elongation at break (EAB) and tensile strength (TS) did not present significant differences among the analyzed films (Table 7). The results for film A (2% alginate), which presented a thickness of 0.046 ± 0.003 mm (Table 4), were lower when compared with Benlloch-Tinoco et al. [44]’s results. They determined the same parameters for the same film, but with a higher thickness of 0.0633 ± 0.0057 mm, and obtained higher EAB and TS, 10.95 ± 2.38% and 62.07 ± 1.80 MPa, respectively, probably due to the film thickness. The same authors observed a decrease in TS and an increase in EAB when the alginate concentration in the film was decreased to 1% and 0.5%.

Some studies suggested that controlling microalgae particle size could improve the quality and mechanical performance of microalgae-based films. Kim et al. [45] showed that reducing the particle size of *Chlorella* sp. through ball milling significantly enhanced the tensile strength of poly(ethylene-vinyl acetate) (EVA) biocomposites. Specifically, the reduction in dried *Chlorella* sp. particles by 72.84% (to 161.43 μm) enabled the production of biocomposites containing 60% (*w*/*w*) microalgae, achieving 61.02% of the tensile strength of pure EVA, comparable to traditional polymers. Additionally, Yang et al. [46] found that the incorporation of microalgae biomass into polymer matrices affected the viscosity and mechanical behavior of the resulting bioplastics, highlighting the importance of achieving homogeneous dispersion to optimize film performance. These studies underscore the critical role of bioactive components and processing techniques in modifying the mechanical properties of microalgae-based films, offering valuable insights for their development in various applications.

#### 3.7.2. Antioxidant Activity of the Films

In general, the addition of all extracts (rich in polysaccharides, protein, and bioactives) to the film formulation enhanced the antioxidant activity of the films (Table 8). Both the films with P-rich extract and the alginate films with PS-rich extract, when combined with bioactive-rich extracts from *Lobosphaera* sp., exhibited higher ABTS and DPPH values than the control film. These findings are consistent with results observed in *Arthrospira* sp. films [17]. However, the ABTS values for P-rich extract films from *Lobosphaera* sp. were lower than those of similar films from *Arthrospira* sp., suggesting that the antioxidant activity of the former microalga is comparatively lower.

Morales-Jimenez et al. [9] found that crude polysaccharide extracts from *Nostoc* sp., *Synechocystis* sp., and *Porphyridium purpureum* either exhibited no antioxidant activity or showed low DPPH and ABTS values. Kontogianni et al. [38] produced a whey protein concentrate film containing 2% Spirulina (*Arthrospira*) biomass and achieved a DPPH radical scavenging value of 35.33 ± 2.76%. They also noted that increasing the biomass concentration in the formulation did not enhance the antioxidant capacity. Similarly, Kia et al. [47] developed a biocomposite film with Zedo gum and sodium caseinate, incorporating *Arthrospira platensis* into the formulation, which significantly increased the antioxidant activity. Notably, lower concentrations of Zedo gum resulted in higher antioxidative capacity.

#### 3.7.3. Antimicrobial Activity of the Films

**Figure 3:**
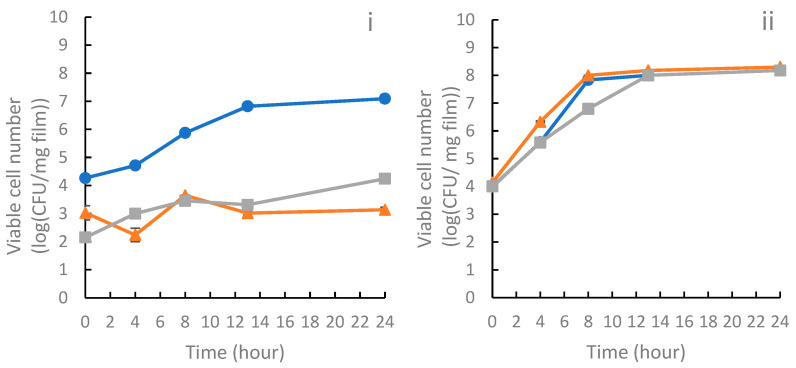
Growth curves of *Listeria monocytogenes* ESB 3562 (**i**) and *Salmonella enterica serovar* Enteritidis ATCC 13076 (**ii**) in contact with films: A (blue line); C (orange line); and E (gray line).

The inclusion of PS-rich extract and bioactive-rich extract from *Lobosphaera* sp. in the film significantly inhibited the growth of *Listeria monocytogenes* (Gram+ bacteria) compared with the control film (alginate). A similar reduction in bacterial growth was observed in the film containing P-rich extract and bioactive-rich extract (Figure 3). In contrast, these extracts did not show any significant effect on the growth of *Salmonella enterica serovar* Enteritidis (Gram-bacteria).

Lopes et al. [21] observed that plant extracts incorporated into alginate films reduced their antibacterial activity against Gram+ and Gram- bacteria. They suggested that chemical interactions between hydroxyl groups in the film and phenolic compounds might contribute to this effect. Additionally, antibiotics are generally less effective against Gram-negative bacteria due to their complex, multi-layered cell wall structure, which hinders the penetration of active compounds [48]. This could explain why the films incorporating bioactive-rich extracts did not inhibit the growth of *Salmonella enterica serovar* Enteritidis (Gram-) in the present study.

The small difference observed between the effects of the films with PS-rich and P-rich extracts on the growth curves of *Listeria monocytogenes* (Figure 3) suggests that the antimicrobial activity of these films against this bacterium is likely attributed to the bioactive extracts. Amaro et al. [48] explained that this activity is linked to lipids present in the extract, such as eicosapentaenoic acid, which is predominantly found as a polar lipid species in structural cell components. They also mention that high levels of palmitoleic acid were detected in *Porphyridium tricornutum* microalgae, and this fatty acid is known to be active against various Gram+ human pathogens. Both of these acids were present in the bioactive-rich extract from *Lobosphaera* sp. in the present study (Table 2). Several scientific studies have demonstrated that certain lipids, such as EPA and other unsaturated fatty acids, can inhibit *Listeria monocytogenes*. Borreby et al. [49] demonstrated that sub-inhibitory concentrations of EPA downregulated the expression of key virulence factors in these strains, indicating that EPA can reduce the pathogenic potential of *Listeria monocytogenes*. Wang and Johnson [50] found that certain unsaturated fatty acids, including C18:3 (alpha-linolenic acid), exhibited strong bactericidal activity against *Listeria monocytogenes* at concentrations of 10 to 20 μg/mL. This suggests that specific fatty acids can effectively inhibit the growth of *Listeria monocytogenes*. Denton et al. [51] assessed the minimum inhibitory concentrations of various unsaturated fatty acids against 63 strains of *Listeria* species. The findings indicated that the inhibitory properties of these fatty acids increased with the degree of unsaturation, suggesting that unsaturated fatty acids are effective in inhibiting *Listeria* species.

### 3.8. Film Application Results

#### 3.8.1. Strawberry Moisture Loss

**Figure 4:**
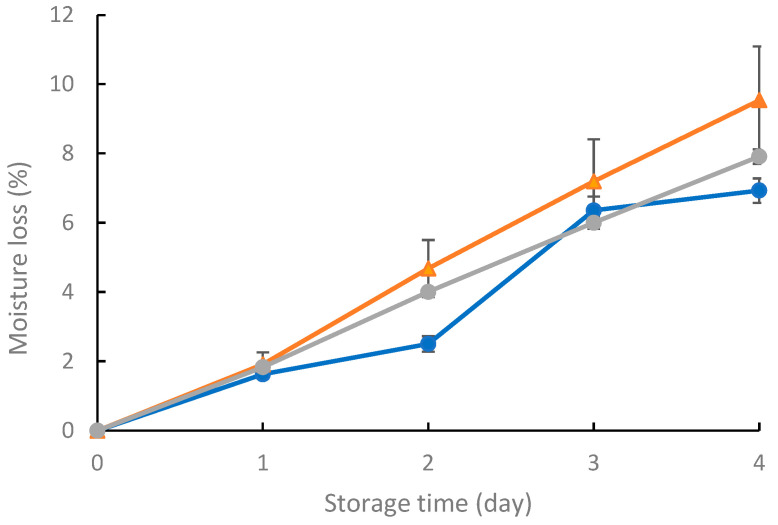
Moisture loss of strawberries stored in flasks closed with film A (blue line); film C (orange line); and film E (gray line).

The moisture content of strawberries increased over time for all films (Figure 4). Similar results were reported by Zhou et al. [52] for alginate films and alginate films containing cinnamaldehyde and xanthan gum. In the present study, after four days, the moisture loss was: 7.91 ± 0.21% for film E, 9.53 ± 1.55% for film C, and 6.93 ± 0.35% for the control film (A). These differences were not statistically significant. However, on day 2, the difference between the films was found to be statistically significant, and the strawberries stored in the flasks closed with film A presented the lowest moisture loss. This could be attributed to the sample variability.

#### 3.8.2. Mesophilic Aerobic Bacteria, Molds, and Yeasts Counts in Strawberry

The microbiological results indicate that films containing the bioactive extract inhibited microbial growth (mesophilic aerobic bacteria, molds, and yeasts) in strawberries (Table 9). The findings confirm that molds and yeasts proliferated more in the control film than in films containing *Lobosphaera* sp. extracts, with a statistically significant difference. For mesophilic aerobic bacteria, the difference in bacterial growth between film A and film E was statistically significant. However, when comparing film A with film D, the difference was not statistically significant.

Martins et al. [53] investigated the application of a 90% hydroalcoholic extract from exhausted olive oil pomace in an alginate coating on strawberries. Their study reported a statistically significant difference in the growth of mesophilic aerobic bacteria and molds and yeasts between coated and uncoated strawberries over a 10-day refrigerated storage period at 4 °C. The antimicrobial activity of microalgae in food applications was evaluated by Shirai et al. [54], who incorporated chitosan, natamycin, *Chlorella vulgaris*, and *Spirulina platensis* into coatings applied to calf fillets stored at 4 °C for 28 days. Their results indicate that, in the control sample, the total bacterial count exceeded acceptable consumption limits after seven days, whereas samples containing microalgae extracts maintained microbial levels below the acceptable limit for the entire 28-day storage period.

## 4. Conclusions

The present study successfully extracted bioactive compounds from *Lobosphaera* sp., demonstrating strong antioxidant activity, high nutritional quality in terms of fatty acid composition, and no cytotoxic effects on Caco-2 cells. Additionally, polysaccharides and proteins were extracted. Biodegradable films incorporating these extracts were successfully developed, exhibiting favorable physical properties such as complete solubility in water, indicating their high biodegradability. The use of protein and bioactive compounds from microalgae extracts proved to be a promising strategy for decreasing the permeability of alginate films, improving their suitability for applications requiring controlled barrier properties. The incorporation of the bioactive extract in the film, in combination with the polysaccharides-rich (PS-rich) or protein-rich (P-rich) extracts, did not affect its mechanical properties, namely elongation at break and tensile strength. The films exhibited strong antioxidant activity, as assessed by ABTS and DPPH assays, with the most effective formulation comprising alginate (2%), P-rich extract (0.5%), and bioactive-rich extract (0.25%). Furthermore, the films demonstrated antimicrobial activity against *Listeria monocytogenes*, primarily due to the bioactive-rich extract. In addition, they were shown to reduce mold and yeast growth in strawberries, promising to extend the shelf life of this produce.

These findings highlight the potential application of films containing *Lobosphaera* sp. extracts as soluble packaging for individual food portions and as active packaging materials designed for controlled antioxidant and antimicrobial release. Future research should be conducted to test the functionality of films from *Lobosphaera* sp. with different fruits and vegetables or fresh-cut produce.

## Figures and Tables

**Table 1:** Film formulation.

Film Formulation	Code
Alginate (2% *w*/*v*)	A
Alginate (2% *w*/*v*) + PS-rich extract (0.5% *w*/*v*)	B
Alginate (2% *w*/*v*) + PS-rich extract (0.5% *w*/*v*) + bioactive-rich extract (0.25% *w*/*v*)	C
Alginate (2% *w*/*v*) + P-rich extract (0.5% *w*/*v*)	D
Alginate (2% *w*/*v*) + P-rich extract (0.5% *w*/*v*) + bioactive-rich extract (0.25% *w*/*v*)	E

**Table 2:** Fatty acids profile in the bioactive-rich extract.

Fatty Acids	Chain Length	Quantity (mg/g)
Butyric acid	C4	0.195 ± 0.005
Caproic acid	C6	0.066 ± 0.003
Caprylic acid	C8	0.160 ± 0.013
Capric acid	C10	0.118 ± 0.012
Lauric acid	C12	0.456 ± 0.086
Myristic acid	C14	0.495 ± 0.150
Myristoleic acid	C14:1	0.674 ± 0.104
Pentadecylic acid	C15	7.884 ± 2.349
*cis*-10-Pentadecenoic Acid	C15:1 *c*10	0.725 ± 0.156
Palmitic acid	C16	443.900 ± 129.518
Palmitoleic acid	C16:1 *c*9	0.645 ± 0.006
Margaric acid	C17	8.258 ± 8.060
*cis*-10-Heptadecenoic acid	C17:1 *c*10	0.880 ± 0.321
Stearic acid	C18	12.772 ± 0.190
Elaidic acid	C18:1 *t*9	0.202 ± 0.042
Oleic acid	C18:1*c*9	200.459 ± 50.139
Linolelaidic acid (ω-6)	C18:2 *t*6	5.180 ± 1.190
Linoleic acid (ω-6)	C18:2 *c*6	110.241 ±110.119
Arachidic acid	C20	1.717 ± 0.421
γ-linolenic acid (ω-6)	γ C18:3	187.497 ± 50.972
Gondoic acid	C20:1	3.410 ± 1.241
α-linolenic acid (ω-3)	α C18:3	5.048 ± 1.014
Heneicosylic acid	C21	1.218 ± 0.166
Eicosadienic acid	C22:2 *c*11 *c*14	1.314 ± 0.272
Behenic acid	C22	2.507 ± 0.388
Eicosadienoic acid (ω-6)	C20:3 *c*8 *c*11 *c*14	3.258 ± 1.279
Erucic acid	C22:1 *c*13	6.567 ± 2.094
Eicosapentaenoic acid (ω-3)	C20:5	2.354 ± 0.074
Tricosylic acid	C23	4.091 ± 1.294
*cis*-13,16-Docosadienoic acid	C22:2	0.807 ± 0.001
Lignoceric acid	C24	1.033 ± 0.326
Nervoic acid	C24:1	2.086 ± 0.219
Docosahexaenoic acid	C22:6	2.826 ±0.994
Σ fat acids		1019.0456 ± 363.151
Σ SFA		485.414 ± 143.042
Σ MUFA		215.648 ± 54.226
Σ PUFA		318.526 ± 165.953
Σ Mufa + Σ PUFA		534.174 ± 220.179
Σ PUFA ω-3		10.228 ±2.114
Σ PUFA ω-6		306.176 ±163.561
AI		0.890 ± 0.123
TI		1.649 ± 0.206
HH		3.750 ± 0.003
HPI		1.151 ± 0.156

**Table 3:** Total phenolic content and antioxidant activity (ABTS, DPPH, ORAC) of the bioactive-rich extract.

TPC	ABTS	DPPH	ORAC
(mg GAE/100 mg DW)	(µmol TE/100 mg DW)		
1.07 ± 0.05	2.44 ± 0.27	1.67 ± 0.15	11.90 ± 1.22

**Table 4:** Thickness, water vapor transmission rate (WVTR), and water vapor permeability (WVP) of the films.

Film	Thickness (mm)	WVPR (g·m^−2^·day^−1^)	WVP (g·mm·m^−2^·day^−1^·kPa^−1^)
A	0.046 ± 0.003 ^d^	575.99 ± 15.50 ^bc^	23.45 ± 1.47 ^bc^
B	0.060 ± 0.003 ^c^	660.93 ± 16.49 ^c^	26.67 ± 0.26 ^c^
C	0.068 ± 0.007 ^bc^	483.09 ± 39.20 ^b^	20.99 ± 2.17 ^abc^
D	0.075 ± 0.003 ^ab^	470.61 ± 17.87 ^b^	19.21 ± 2.80 ^ab^
E	0.084 ± 0.003 ^a^	271.10 ± 19.84 ^a^	12.27 ± 0.98 ^a^

Different letters in each number mean significant differences (*p* < 0.05).

**Table 5:** Color parameters of the films.

Film	L*	a*	b*	Hue (°)	Chroma
A	51.79 ± 3.77 ^c^	2.30 ± 0.15 ^b^	0.93 ± 0.11^d^	21.85 ± 1.32 ^d^	2.48 ± 0.18 ^d^
B	59.15 ± 9.03 ^bc^	2.24 ± 0.45 ^b^	7.72 ± 0.92 ^c^	73.32 ± 2.49 ^b^	7.78 ± 0.97 ^c^
C	70.43 ± 8.64 ^ab^	0.34 ± 0.05 ^c^	12.98 ± 1.73 ^b^	88.46 ± 0.42 ^a^	12.98 ± 1.72 ^b^
D	85.71 ± 6.02 ^a^	3.7 ± 0.33 ^a^	4.95 ± 0.47 ^c^	53.19 ± 0.71 ^c^	6.18 ± 0.57 ^c^
E	54.06 ± 7.80 ^c^	−0.95 ± 0.27 ^d^	17.47 ± 2.66 ^a^	−86.96 ± 0.61 ^e^	17.49 ± 2.67 ^a^

Different letters in each column mean significant differences (*p* < 0.05).

**Table 6:** Solubility of the films.

Film	H_2_O	Acetic Acid 3%	EtOH 10%	EtOH 20%	EtOH 50%
A	100	16.9 ± 1.6 ^b^	100	100	100
B	100	13.6 ± 1.5 ^b^	100	100	100
C	100	17.3 ± 1.8 ^b^	100	100	100
D	100	35.2 ± 6.8 ^a^	100	100	100
E	100	32.0 ± 2.5 ^a^	100	100	100

Different letters in each column mean significant differences (*p* < 0.05).

**Table 7:** Elongation at break (EAB) and tensile strength (TS) of the films.

Film	Elongation at Break (%)	Tensile Strength (MPa)
A	4.97 ± 1.99 ^a^	31.19 ± 6.63 ^a^
C	5.23 ± 1.92 ^a^	32.00 ± 5.65 ^a^
E	4.08 ± 0.84 ^a^	28.77 ± 6.33 ^a^

Different letters in each column mean significant differences (*p* < 0.05).

**Table 8:** Antioxidant activity of the films.

Film	ABTS	DPPH
	(µM TE/mg Film)	
A	120.15 ± 6.81 ^c^	85.97 ± 3.19 ^c^
B	260.67 ± 31.94 ^b^	111.34 ± 16.63 ^bc^
C	394.61 ± 35.69 ^a^	153.15 ± 20.07 ^b^
D	369.62 ± 73.71 ^a^	115.39 ± 13.81 ^bc^
E	451.06 ± 14.68 ^a^	212.81 ± 39.12 ^a^

Different letters in each column mean significant differences (*p* < 0.05).

**Table 9:** Mesophilic aerobic bacteria, molds, and yeasts counts in strawberries stored in flasks closed with films A, C, and E.

Film	Mesophilic Aerobic Bacteria (log (CFU/g Strawberry))	Molds and Yeasts (log (CFU/g Strawberry))
A	2.29 ± 2.14 ^b^	2.33 ± 0.24 ^b^
C	1,77 ± 1.81 ^ab^	1.11 ± 0.40 ^a^
E	1.34 ± 1.05 ^a^	0.63 ± 0.34 ^a^

Different letters in each column mean significant differences (*p* < 0.05).

## Data Availability

The original contributions presented in the study are included in the article, further inquiries can be directed to the corresponding authors.
